# Diffusion-weighted imaging in monitoring the pathological response to neoadjuvant chemotherapy in patients with breast cancer: a meta-analysis

**DOI:** 10.1186/s12957-018-1438-y

**Published:** 2018-07-18

**Authors:** Wen Gao, Ning Guo, Ting Dong

**Affiliations:** 1 Department of Trauma Surgery, Tianjin Fourth Central Hospital, No.1 Zhongshan Road, Hebei District, Tianjin, 300010 China; 2 Department of Breast Surgery, Tianjin Fourth Central Hospital, No.1 Zhongshan Road, Hebei District, Tianjin, 300010 China; 3 Department of Cardiovascular Medicine, Guizhou Provincial People’s Hospital, No. 83 Zhongshandong Road, Guiyang City, 550002 Guizhou China

**Keywords:** Neoadjuvant chemotherapy, Breast cancer, Diffusion-weighted imaging, Pathological response, Meta-analysis

## Abstract

**Background:**

Diffusion-weighted imaging (DWI) is suggested as an non-invasive and non-radioactive imaging modality in the identification of pathological complete response (pCR) in breast cancer patients receiving neoadjuvant chemotherapy (NACT). A growing number of trials have been investigating in this aspect and some studies found a superior performance of DWI compared with conventional imaging techniques. However, the efficiency of DWI is still in dispute. This meta-analysis aims at evaluating the accuracy of DWI in the detection of pCR to NACT in patients with breast cancer.

**Methods:**

Pooled sensitivity, specificity, and diagnostic odds ratio (DOR) were drawn to estimate the diagnostic effect of DWI to NACT. Summary receiver operating characteristic curve (SROC), the area under the SROC curve (AUC), and Youden index (*Q) were also calculated. The possible sources of heterogeneity among the included studies were explored using single-factor meta-regression analyses. Publication bias and quality assessment were assessed using Deek’s funnel plot and QUADAS-2 form respectively.

**Results:**

Twenty studies incorporated 1490 participants were enrolled in our analysis. Pooled estimates revealed a sensitivity of 0.89 (95% CI, 0.86–0.91), a specificity of 0.72 (95% CI, 0.68–0.75), and a DOR of 27.00 (95% CI, 15.60–46.73). The AUC of SROC curve and *Q index were 0.9088 and 0.8408, respectively. The results of meta-regression analyses showed that pCR rate, time duration of study population, and study design were not the sources of heterogeneity.

**Conclusion:**

A relatively high sensitivity and specificity of DWI in diagnosing pCP for patients with breast cancer underwent NACT treatment was found in our meta-analysis. This finding indicated that the use of DWI might provide an accurate and precise assessment of pCR to NACT.

## Background

Neoadjuvant chemotherapy (NACT), since its first appearance until nowadays, has become a standard therapy for patients with breast cancer. It is suggested to have beneficial effect especially on locally advanced or inflammatory breast cancer [1]. The major benefit of NACT is to reduce the tumor size and to downstage the tumor burden, which may lead to the successful performance of breast-conserving surgery instead of mastectomy [2]. In addition, assessing the treatment responses to NACT can also help to determine the right time to perform the operation or to adjust the therapy regimen in case of an unfavorable tumor response at an early stage [3]. It is well-established in some previous studies that the response to NACT is correlated with long-term outcomes for breast cancer patients. Studies also reveal that pathological complete response (pCR) patients may have a superior chance to achieved disease-free survival and overall survival [4–6]. Nevertheless, only a minority of patients were featured with pCR due to the heterogeneity of breast cancer. We could not accurately observe the pCR until the definitive breast surgery was completed, which always led to inappropriate surgery decision-making for patients [7, 8]. Therefore, it is crucial to find an effective method to separate the patients who have achieved pCR from pathological non-responders (pNR) before surgery.

Mammography, ultrasonography, positron emission tomography-computed tomography (PET/CT) and magnetic resonance imaging (MRI) are the most commonly applied conventional imaging techniques for the detection of NACT responses. Previous studies found that MRI was superior to mammography or ultrasonography in evaluating therapeutic response of NACT in breast cancer [9, 10]. A meta-analysis demonstrated a higher sensitivity in PET/CT and a higher specificity in MRI for the assessment of pCR [11]. Currently, contrast-enhanced magnetic resonance imaging (DCE-MRI) is frequently and commonly used for tumor response evaluation after NACT. However, the information provided by DCE-MRI regarding blood flow and vessel permeability might cause difficulty in differentiating viable residual cancer form surrounding scar, necrosis, fibrosis, or reactive inflammation resulting from NACT response. Thus, DCE-MRI has deficiencies for the examination of pathological response to NACT [12].

Diffusion-weighted imaging (DWI), with its unique tissue contrast mechanism, is regarded as a potential modality to overcome the limitations of traditional DCE-MRI evaluation [13]. DWI reveals the thermally driven motion of water molecules in the target tissue. It offers information concerning the integrity of cell membranes and cancer cellularity. The apparent diffusion coefficient (ADC), which can be quantified and measured on DWI, represents the complex diffusion of water in tissues [14]. With those characteristics, DWI can be sensitive in detecting the changes in the intratumor induced by NACT [15].

The accuracy of conventional imaging modality including MRI, PET/CT, mammography, and ultrasonography in the assessment of the pCR to NACT has been investigated by several recent meta-analyses [16–19]. However, no previous study has focused on analyzing the performance of DWI in detecting the pCR in breast cancer to NACT systematically. In researches providing DWI evaluation, the data were limited. They only involved a small amount of studies which might weaken the statistic power of the analysis. By combining all available data, the present meta-analysis intended to evaluate the diagnostic role of DWI in monitoring pCR in breast cancer to NACT.

## Method

### Literature search

Databases PubMed and EMBASE were systematically searched from database inception to August 2017 for all the potential publications. Articles in regard to DWI assessing tumor response in patients with breast cancer underwent NACT treatment were retrieved using the following search terms: “diffusion-weighted imaging” or “DW-MRI” or “DWI,” “breast cancer” or “breast tumor” or “breast,” “response” or “prediction,” “neoadjuvant chemotherapy” or “chemotherapy” or “NACT,” “diagnosis” or “accuracy” or “performance.” One reviewer screened all the titles and abstracts for eligibility. The remaining studies after removing the duplications and non-related articles were examined in full text by a second reviewer. Reference list of the enrolled studies and other meta-analyses were searched manually for any additional publication that was not included in the original search. Articles published in English and Chinese were eligible for inclusion.

### Eligibility criteria

Studies were considered as usable if they met the following criteria: (1) patients were diagnosed with breast cancer and received NACT treatment; (2) DWI scan should be performed before and during (after) NACT; (3) studies provided available data of true positive (TP), true negative (TN), false positive (FP), false negative (FN), sensitivity and specificity findings, either directly or indirectly; (4) studies that with different additional surgery or other adjunctive treatment were all considered available. We excluded studies with inseparable combined data of different diagnostic methods, duplicated articles, reviews, case reports, and other non-related studies.

### Data extraction

The following information were extracted in the process of full-text review of the eligible studies: first author, region where the study took place, year of publication, patients’ demographic (sample size, gender, age) and clinical characteristics (disease stages, histologic subtype), chemotherapeutic regimens used in NACT, cycles of NACT, image interpretation (blinded or not), magnet strength of DWI, timing of DWI evaluation, applied surgery after NACT, reference standard of pathologic response, definition of pCR, and number of complete responders and non-responders. The number of TP, TN, FP, and FN was obtained from the pathological results of the DWI scan. Two independent reviewers carried out the data extraction process, and discrepancies were solved by discussion till consensus was reached.

#### Quality assessment

The updated version of quality assessment of diagnosis accuracy study form (QUADAS-2) was used in the assessment of methodological quality of the enrolled studies. This appliance was specifically developed for systematic review and meta-analysis of diagnostic accuracy studies [20]. The QUADAS-2 test contains four aspects of questions: patients’ selection, index text, reference standard, and flow and timing. Risk of bias was assessed in all fields, and the concerns regarding applicability was evaluated in the first three domains. The signaling questions of each key domain can help in judging studies as having high, low, or unclear risk.

### Statistical analysis

A 2-by-2 contingency table separating patients into TP, TN, FP, and FN groups was constructed for each enrolled study. Based on this table, sensitivity and specificity were calculated. Diagnostic odds ratio (DOR) was measured to estimate the effectiveness of DWI by calculating the odds of achieving pCR in patients with a positive test result to patients with a negative test result. The area under the curve (AUC) of the summary receiver operating characteristic curve (SROC) was calculated to measure the performance of DWI. An AUC close to 1 indicates a favorable diagnostic performance, whereas a close to 0.5 AUC implies a poor test result. The Youden index (*Q), which is used in conjunction with SROC analysis and recognized as a preferred statistic to reflect the diagnostic value, were also assessed. A *Q index of 1 indicates a perfect test result. All data analyses were carried out using statistical software package Meta-DiSc 1.4 and Stata version 15.0.

The heterogeneity among studies was evaluated with chi-squared test and *I*^2^ statistics. A random effects model was used for outcome estimation if *I*^2^ < 50%, and a fixed effects model was chosen if *I*^2^ > 50%. Threshold effect was one of the important sources of heterogeneity in diagnostic accuracy test. The Spearman correlation coefficients can determine the existence of threshold effect. It indicated no threshold effects among studies if *P* value > 0.05. Then, the bivariate mixed-effect models were used to draw the forest plot and SROC. In addition, heterogeneity caused by non-threshold effect was also explored utilizing single-factor meta-regression analyses. We separated the studies into different subgroups, in terms of pCR rate (mean = 21%), the duration of the study population (midpoint = 2009), and whether the image interpretation was blinded. Variances were considered as sources of heterogeneity if their regression coefficients reached statistical significance (*P* < 0.05). Publication bias was analyzed using Deeks’ funnel plot and an asymmetry test. The absence of a non-zero slope coefficient (*P* > 0.05) indicates no publication bias exists among the included studies.

## Results

### Study selection

**Fig. 1:**
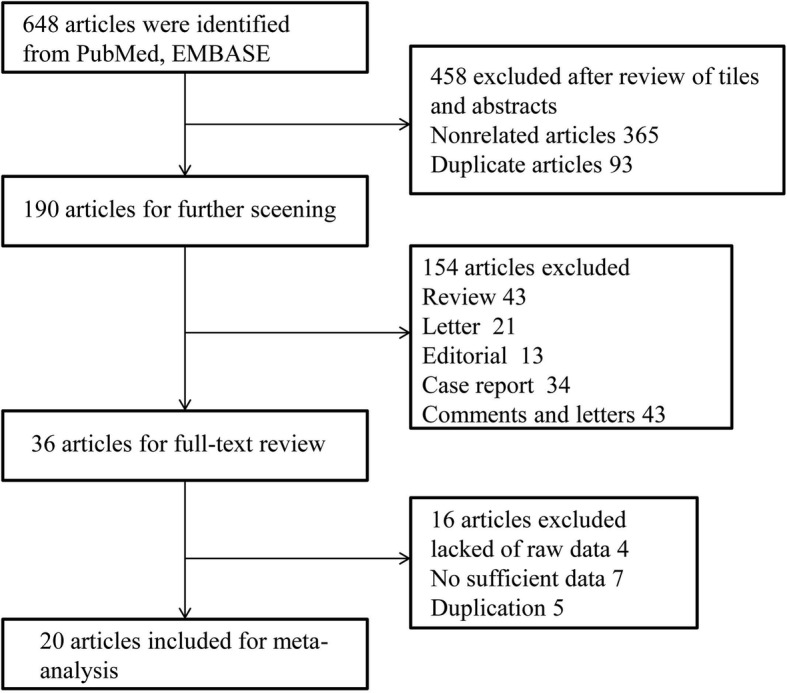
Flow diagram of literature search

The systematic search and manual cross-checking of references yielded 648 articles in total from PubMed and EMBASE database initially. After excluding the obviously irrelevant articles according to titles and abstracts, 190 remained as potential candidates for inclusion. One hundred fifty-four articles were further ruled out as they disagreed with our inclusion criteria. An additional of 16 articles were excluded after careful full-text review. The reasons for exclusion were as follows: studies lacked of raw data (*n* = 4); the provided data were not sufficient to construct or calculate the contingency table (*n* = 7); studies presented repetitive data from author with additional studies (*n* = 5). Eventually, 20 studies were enrolled in the analysis. Figure 1 presented the procedure of literature search and study selection.

### Study description

The included 20 studies consisted a total of 1490 patients [7, 12, 21–38]. The sample size ranged from 28 to 225 (median 75) patients. Thirteen of the enrolled studies used a 1.5 T magnet strength and 6 used 3 T for measurement. The study of Mani et al. [30] did not provide information about the applied magnet strength. In more than a half of the studies, radiologists were blinded to the pathological data. The basic information of each included trail were described in Table 1. The classifications used to identify pathologic response after NACT were varied from study to study. Three studies utilized Miller-Payne grading system, another three studies applied Mandard’s tumor regression grade (TRG) criteria, one study used a Japanese Breast Cancer Society criteria, and one used the Chevalier-Sataloff classifications. The remaining articles applied a standard set by the researchers. Therefore, the definition of pCR after NACT of each study was not identical. Patients who reached Miller-Payne grade V, TRG 1, Japanese Breast Cancer Society grade 3, and Chevalier class 1, Sataloff A were classified as pCR in studies using the above criteria. Of the other 12 studies, 5 of them considered patients with no residual invasive cancer in the breast or lymph nodes as achieving pCR. Five studies defined pCR as the absence of invasive cancer and two studies defined pCR as the disappearance of recognizable invasive tumor cells but ductal carcinoma in situ (DCIS) may have been present. Breast-conserving surgery or mastectomy was performed in nine studies and lumpectomy or mastectomy was conducted in two studies. Four studies declared that patients received surgery after NACT treatment, but they did not clarify the type of surgery. The final five studies did not mention surgery after NACT. As for NACT regimens, patients in the same study or in different studies received diverse chemotherapy. The detailed information of NACT treatment of the enrolled literature were presented in Table 2.

**Table 1:** Basic characteristics of included studies

Study	Year	Study design	No. of cases	Age (mean range)	Disease stages	Histologic subtype	Magnet strenth (T)	Duration of the patients (years, month)	Blind	Timing of evaluation
Agarwal	2017	NR	38	44.2(19–65)	LABC, stage II/III	IDC/DCIS	1.5 T	NR		Pre-NAC and after 1.3 cycles
Atuegwu	2013	NR	28	44.9 (28–67)	Stage II/III	NR	3.0 T	NR		Pre-NAC and after 1 cycle, and post-NAC
Belli	2011	Pro	51	48.4 (26–66)	NR	IDC/ILC	1.5 T	2007.01–2009.01	Blind	Pre-NAC and post-NAC within 4 weeks
Bufi	2014	Retro	225	47 (26–67)	Stage II/III	IDC/ILC	1.5 T	2007–2012	Blind	Pre-NAC and post-NAC within 4 weeks
Bufi	2015	Retro	225	47 (26–67)	LABC, stage II/III/IV	IDC/ILC	1.5 T	2007–2012	Blind	Pre-NAC and post-NAC within 4 weeks
Che	2016	NR	36	50.9 (27–75)	LABC	IDC/ILC	3.0 T	2014.03–2015.05	Blind	Pre-NAC and after 2 cycles
Fangberget	2010	Pro	31	50.7 (37–72)	Stage II/III/IV	IDC/ILC	1.5 T	2007.04–2008.10	Blind	Pre-NAC and after 4 cycles, and post-NAC
Fujimoto	2013	NR	56	50.9(27–70)	Stage II/III	IDC	1.5 T	2006.02–2009.12	Blind	Pre-NAC and post-NAC within 3 weeks
Li	2011	Pro	32	46 (25–63)	LABC	NR	1.5 T	2007.07–2010.07		Pre-NAC and after 1 cycle
Li	2015	Pro	42	46.8 (28–67)	Stage II/III	NR	3.0 T	NR		Pre-NAC and after 1 cycle, post-NAC
Luo	2014	Retro	71	46.1 (29–72)	NR	IDC	3.0 T	2010.03–2012.12	Blind	Pre-NAC, after 2 cycles and post-NAC
Mani	2013	NR	28	45 (28–67)	Stage II/III	NR	NR	NR		Pre-NAC, after 1 cycle and post-NAC
Study	Year	Study design	No. of cases	Age (mean range)	Disease stages	Histologic subtype	Magnet strenth(T)	Duration of the patients (year, months)	Blind	Timing of evaluation
Park	2010	Retro	53	43.7 (24–65)	Stage II/III	IDC/ILC	1.5 T	2007.03–2008.05	Blind	Pre-NAC and after 3 cycles
Park	2011	Retro	34	44 (27–60)	LABC	IDC/ILC	1.5 T	2007.04–2008.05	Blind	Pre-NAC and after 3–6 cycles
Richard	2013	Retro	118	53.2 (23–83)	LABC, stage II/III/IV	IDC/ILC	1.5 T	2008.07–2011.05	Blind	Pre-NAC and post-NAC less than 2 weeks
Sharma	2009	Retro	56	48.5 (25–75)	LABC	IDC	1.5 T	2003.12–2006.12		Pre-NAC and after 1, 2, 3 cycles
Shin	2012	Retro	90	46 (24–68)	Stage I/II/III	IDC/ILC	1.5 T	2009.01–2011.05		Pre-NAC and post-NAC
Weis	2015	Retro	33	46 (28–67)	Stage II/III	NR	3.0 T	NR		Pre-NAC, after 1 cycle and post-NAC
Woodhams	2010	NR	69	NR	NR	IDC/ILC	1.5 T	2005.01–2008.10	Blind	Pre-NAC, after 4 cycles the post-NAC
Xu	2017	NR	174	45.7 (28–64)	LABC, stage II/III	IDC/ILC	3.0 T	2011.09–2014.12	Blind	Pre-NAC, after 1 cycle and post-NAC

*LABC* locally advanced breast cancer; *IDL* invasive ductal carcinoma; *ILC* invasive lobular carcinoma; *Pro* prospective; *Retro* retrospective; *NR* not reported

**Table 2:** Characteristics of included studies for neoadjuvant chemotherapy

Study	Year	No. of cases	Classification of pathologic response	Definition of pCR	NACT regimens	Surgery after NACT
Agarwal	2017	38	Miller-Payne	Miller-Payne grade V	CEF, CAF, CEF + DE, DE, DC + Herceptin, DEC	Modified radical mastectomy or wide local excision
Atuegwu	2013	28	–	No residual invasive cancer in the breast or lymph nodes	AC + taxol, Taxotere, Taxol + cisplatin ± everolimus, Trastuzumab +carboplatin + ixabepilone, Trastuzumab, and lapatinib	NR
Belli	2011	51	Mandard’s TRG criteria	TRG 1	FEC, AT, TAC, and TC ± carboplatinum or trastuzumab	Surgery
Bufi	2014	225	Mandard’s TRG criteria	TRG 1	Doxorubicin and cyclophosphamide, and taxanes-based regimens	Breast-conserving and nipple sparing surgery; Surgical excision
Bufi	2015	225	Mandard’s TRG criteria	TRG 1	Doxorubicin, taxane, and cyclophosphamide-based regimens	Breast-conserving and nipple sparing surgery; Surgical excision
Che	2016	36	Miller-Payne	Miller-Payne grade V	Paclitaxel with epirubicin or paclitaxel with carboplatin	Breast-conserving surgery with axillary nodal clearance or modified radical mastectomy.
Fangberget	2010	31	–	Absence of invasive cancer	5-fluoro-uracil, epirubicin and cyclophosphamide	Surgery
Fujimoto	2013	56	Japanese Breast Cancer Society criteria	Necrosis or disappearance of all tumor cells	Adriamycin and cyclophosphamide, paclitaxel, 5-fluorouracil, epirubicin, and cyclophosphamide, paclitaxel	Lumpectomy or mastectomy
Li	2011	32	–	Absence of invasive cancer on breast tumor and lymph nodes	Docetaxel and epirubicin	Breast-conserving surgery or modified radical mastectomy
Li	2015	42		No invasive tumor in the breast	DOX + Cyc + Tax, Cis/Tax±RAD001, Tra + Car, Tra/Car/Her, Tax	Mastectomy or lumpectomy
Luo	2014	71	Miller-Payne	Miller-Payne grade V	NR	NR
Mani	2013	28	–	No residual tumor in the breast or lymph nodes	Adriamycin/cytoxan, taxol/trastuzumab; docetaxel, carboplatin, and trastuzumab; or lapatinib and trastuzumab	Surgery
Park	2010	53	–	Absence of recognizable invasive tumor cells (DCIS may have been present)	Docetaxel and doxorubicin with granulocyte colony–stimulating factor	Modified radical mastectomy or breast-conserving surgery
Park	2011	34	–	No residual malignancy and no sign of cancer cells; no residual invasive cancer and DCIS present	Doxorubicin and docetaxel; paclitaxel, gemcitabine and trastuzumab	Modified radical mastectomy or breast-conserving surgery
Richard	2013	118	Chevalier-Sataloff classifications	Chevalier class 1, Sataloff A	Epirubicin and cyclophosphamide, docetaxel; epirubicin and cyclophosphamide, trastuzumab	Mastectomy or breast-conservative surgery
Sharma	2009	56	–	No residual tumor	CEF; PþE	NR
Shin	2012	90	–	No residual tumor or absence of invasive cancer, but presence of DCIS	Doxorubicin and cyclophosphamide; cyclophosphamide and docetaxel; adriamycin plus docetaxel; 5-fluorouracil, epirubicin and cyclophosphamide; trastuzumab plus paclitaxel	Surgery
Weis	2015	33	–	No residual tumor in the breast or nodes	Paclitaxel, carboplatin, and trastuzumab; doxorubicin and cyclophosphamide, paclitaxel; cisplatin and paclitaxel ± everolimus	NR
Woodhams	2010	69	–	No residual disease or no invasive cancer or DCIS present	Anthracycline and cyclophosphamide, paclitaxel	Quadrantectomy or mastectomy
Xu	2017	174	–	No residual tumor in the breast or nodes	Cyclophosphamide + epirubicin and tatotere	NR

*Miller-Payne grade V*, showed complete disappearance of malignant cells at the site of tumor with only vascular fibroelastotic stroma seen with macrophages; *TRG 1*, complete regression, absence of residual tumor cells; *Chevalier class 1*, disappearance of all tumors on either macroscopic or microscopic assessment; *Sataloff A*, total or near total therapeutic effect; *CEF*, cyclophosphamide epirubicin 5-Fluorouracil; *CAF* cyclophosphamide adriamycin 5-fluorouracil; *DE*, docetaxel epirubicin; *DC*, docetaxel cisplatin; *DEC*, docetaxel epirubicin cisplatin; *FEC*, fluorouracil + epirubicin + cyclophosphamide; *AT*, doxorubicin + taxanes; *TAC*, taxanes + doxorubicin + cyclophosphamide; *TC*, taxanes + cyclophosphamide; *Dox*, doxorubicin; *Cyc*, cyclophosphamide; *Cis*, cisplatin; *PþE*, paclitaxel and epirubicin; *NR*, not reported

#### Quality assessment

**Fig. 2:**
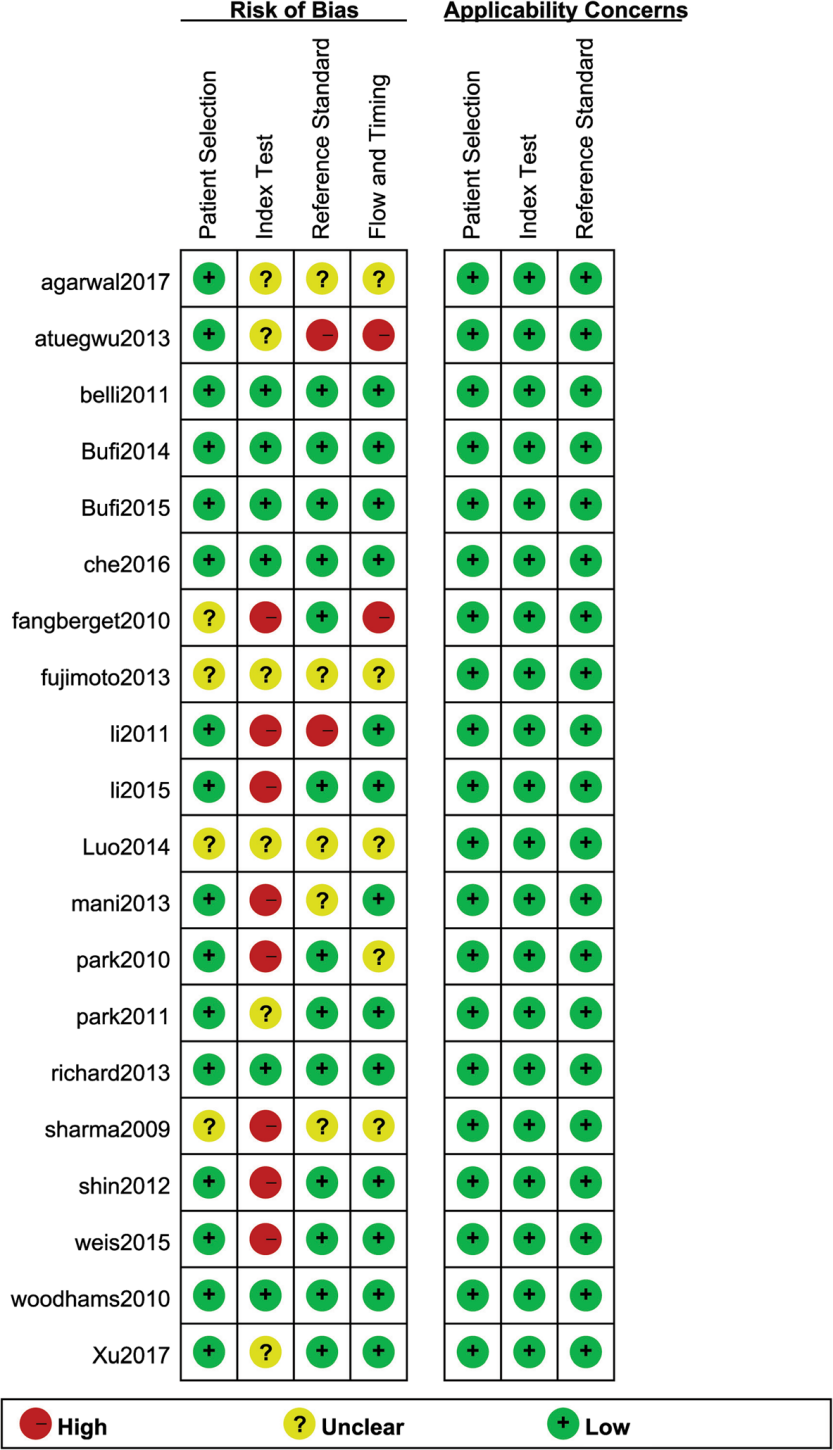
Methodological quality summary of 20 included studies

The result of the QUADAS-2 form revealed that the included studies contained satisfying and eligible qualities. The detailed information and the distribution results of each enrolled study were displayed in Fig. 2.

### Performance of DWI

**Fig. 3:**
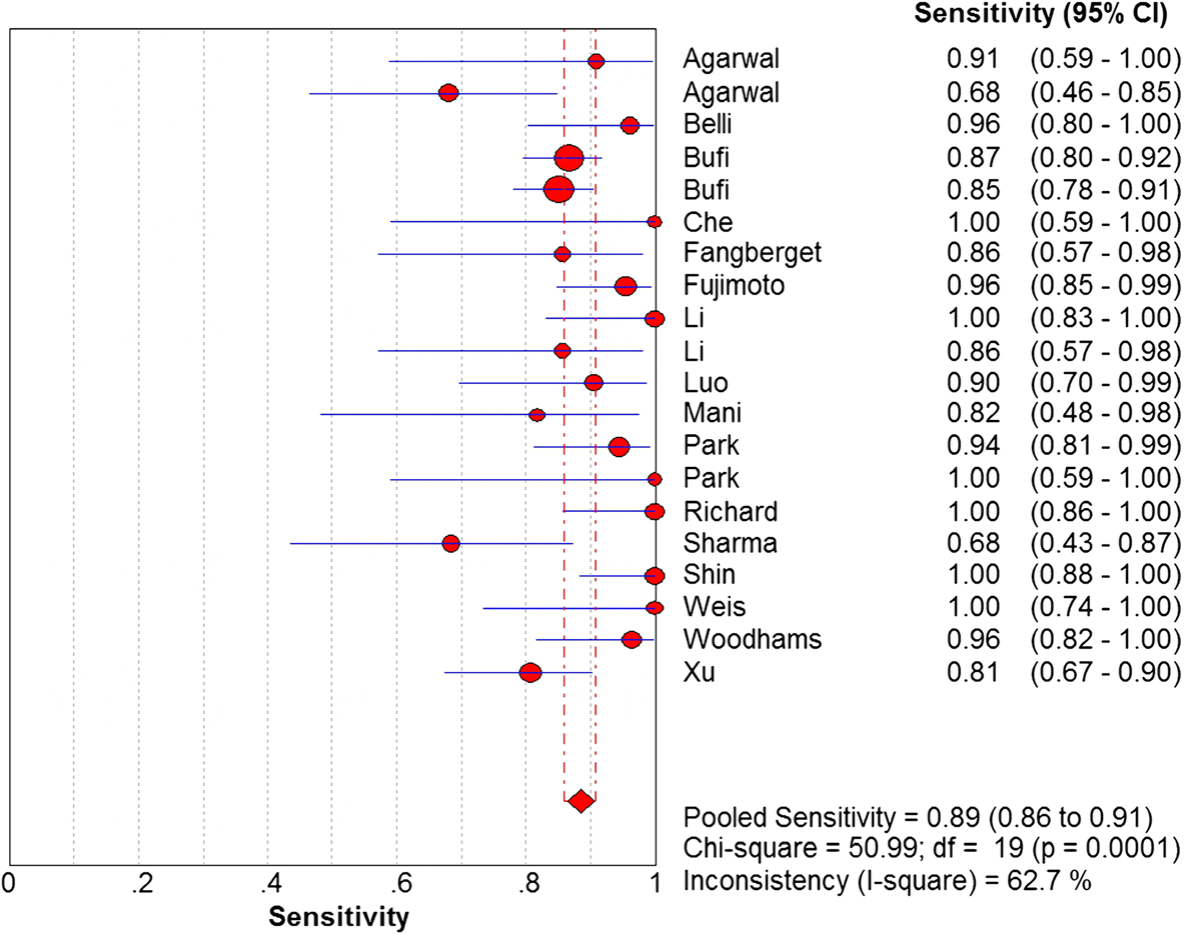
Forest plot of DWI in sensitivity to predict pCR

**Fig. 4:**
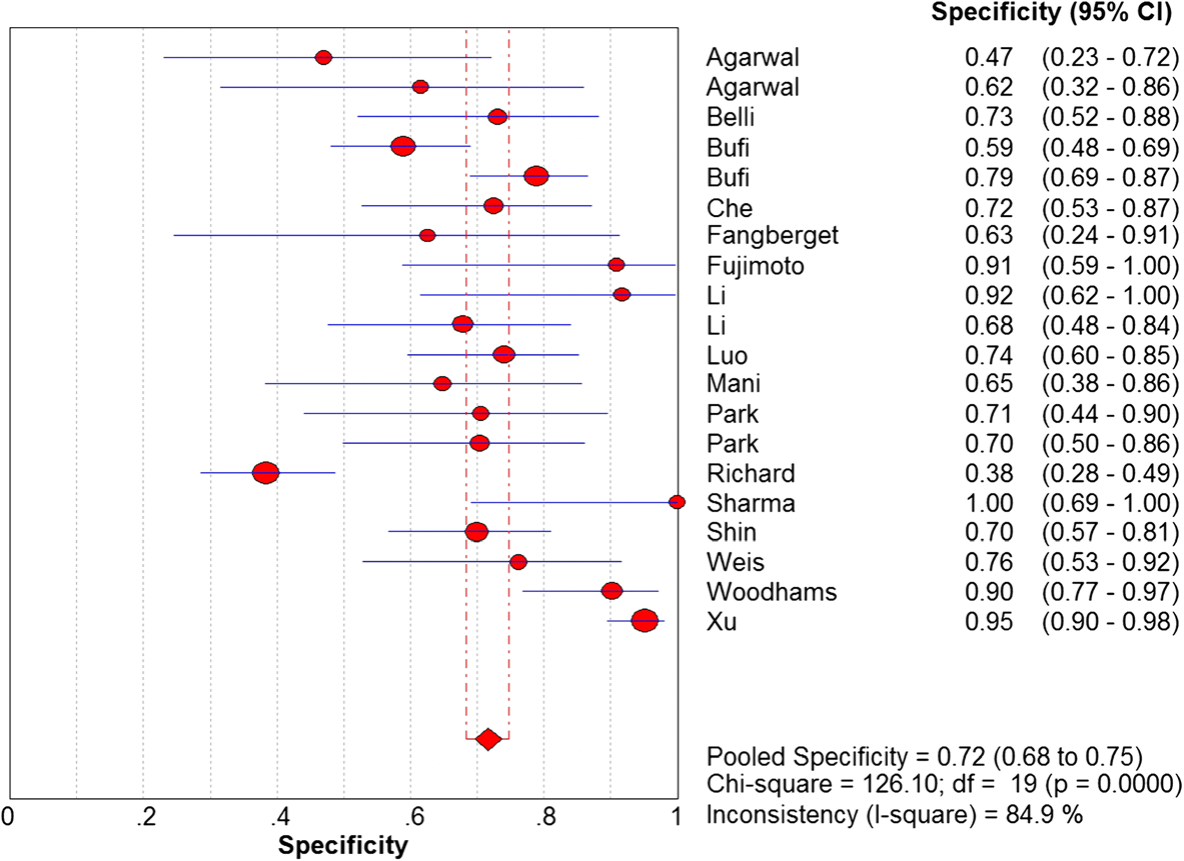
Forest plot of DWI in specificity to predict pCR

**Fig. 5:**
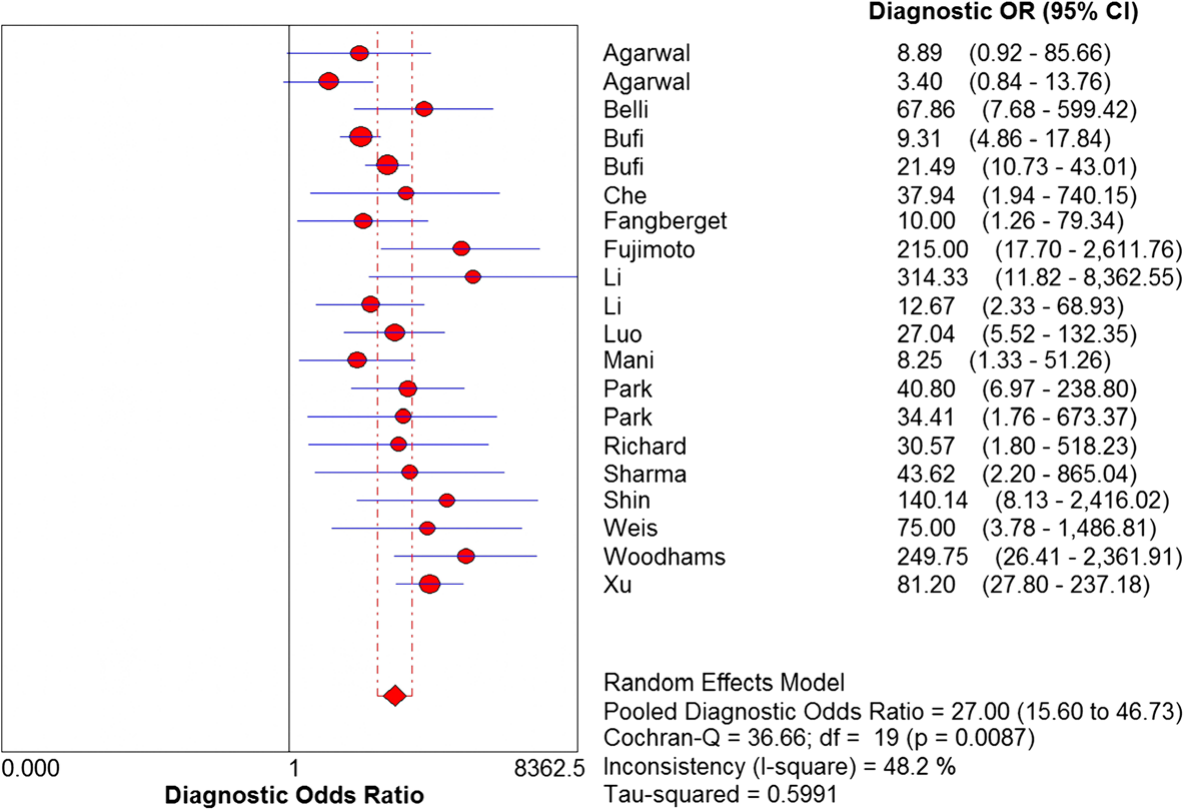
Forest plot of DOR of 20 included studies

**Fig. 6:**
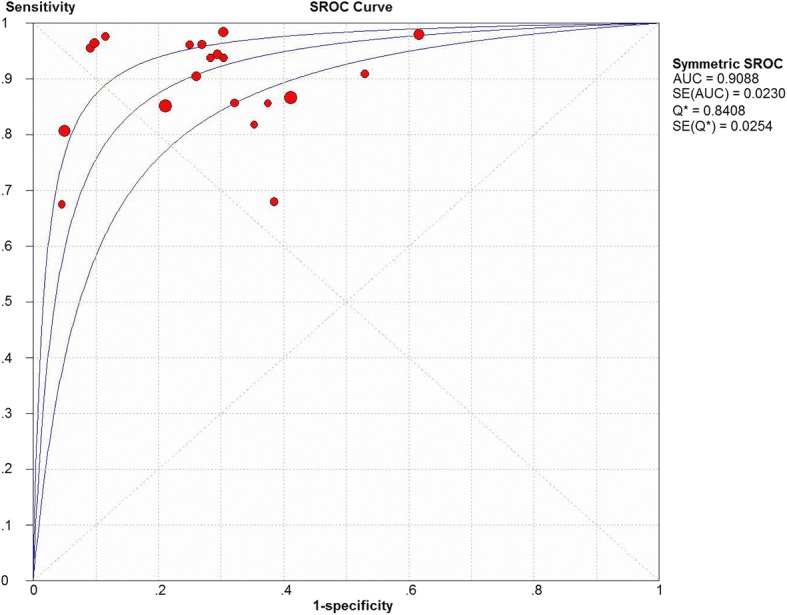
SROC to predict pCR in primary breast cancer by DWI

**Fig. 7:**
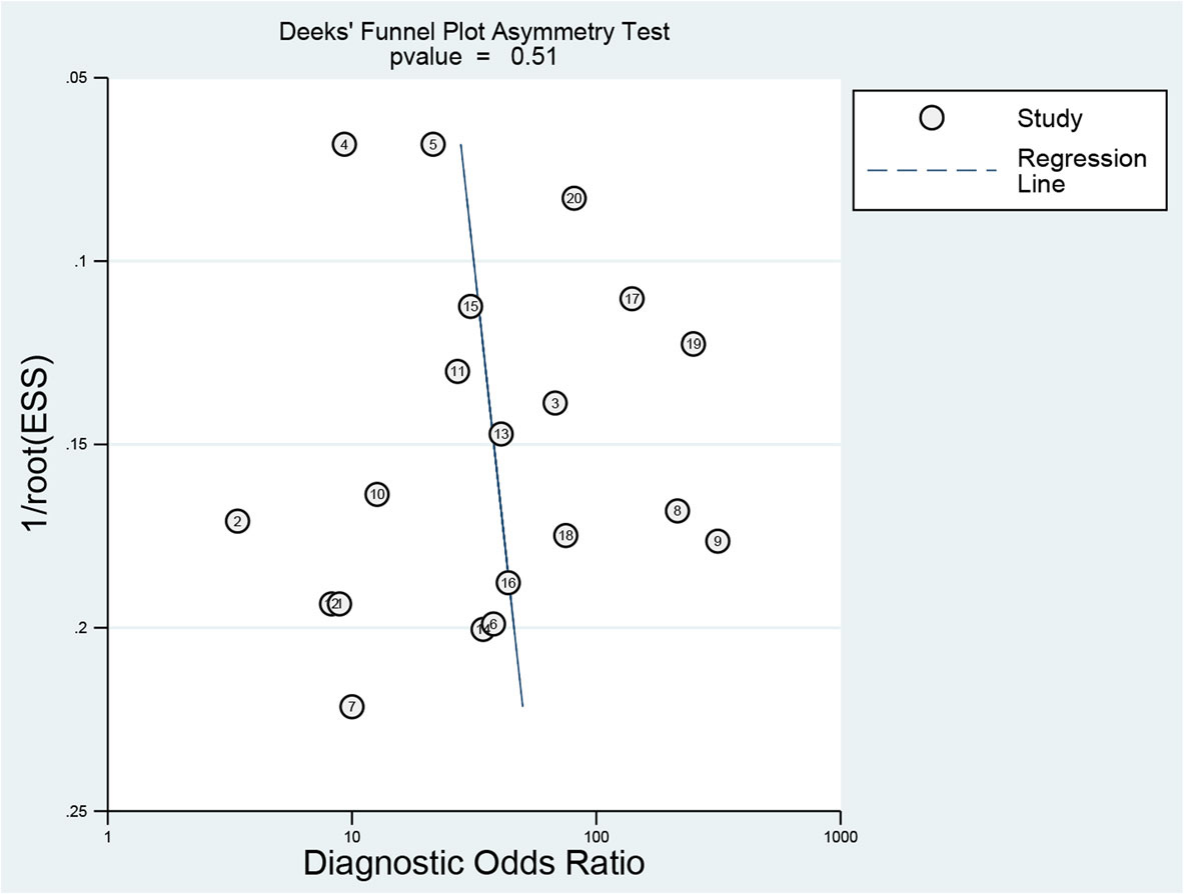
Funnel plot of publication bias

The sensitivity and specificity of all 20 selected studies ranged from 0.68 (95% CI, 0.43–0.87) to 1.00 (95% CI, 0.59–1.00), and from 0.38 (95% CI, 0.28–0.49) to 0.95 (95% CI, 0.90–0.98), respectively. The pooled estimate of 20 studies demonstrated a sensitivity of 0.89 (95% CI, 0.86–0.91) (Fig. 3), a specificity of 0.72 (95% CI, 0.68–0.75) (Fig. 4), and a DOR of 27.00 (95% CI, 15.60–46.73) (Fig. 5). Figure 6 presented the AUC value, which represented the overall diagnostic accuracy of DWI, was 0.9088 ± 0.0230, and the *Q index was 0.8408 ± 0.0254. The outcomes of the analyses suggested that DWI modality was provided with eligible diagnostic performance in the differentiation of NACT responders and non-responders. The publication bias was shown in Fig. 7. Confirming by the Deeks’ funnel plot asymmetry test, no significant publication bias (*P* = 0.51) existed in the present study.

### Heterogeneity text

The statistical results confirmed that there was heterogeneity of DWI both in sensitivity (*I*^2^ = 62.7%) and in specificity (*I*^2^ = 84.9%). The Spearman correlation coefficients’ *P* values (0.565, *P* > 0.05) disclosed the absence of threshold effect in the DWI evaluation.

Single-factor meta-regression analyses were also performed to assess the non-threshold effect. Three subgroups, regarding different pCR rate, treatment duration of patients and whether the researchers were blinded to patients’ therapeutic responses to NACT and pathological findings, were analyzed. Table 3 listed the results of meta-regression analyses. The results demonstrated no statistically significant differences among each subgroup, which indicated that pCR rate, treatment duration, and study design (blinded or not) were not strongly associated with DWI accuracy.

**Table 3:** Results of regression meta-analysis

	Pathologic complete response rate	The duration of the patients	Blind
Coefficient	− 0.314	− 0.365	0.243
Standard error	0.7153	0.7715	0.7349
*P* value	0.6673	0.6427	0.7457
RDOR	0.73	0.69	1.27
[95% CI]	(0.16 to 3.36)	(0.13 to 3.59)	(0.27 to 6.11)

## Discussion

DWI, with its rapid, non-invasive, and without the use of contrast agent characteristic, has emerged as a practical mean to overcome the limitation of DCE-MRI [23, 39]. However, to our knowledge, no previous meta-analysis focused on evaluating the diagnostic performance of DWI in detecting patients’ complete response to NACT in breast cancer. Thus, we designed the current meta-analysis specific for this purpose. By combining data from 20 studies, we detected a 0.89 sensitivity and a 0.72 specificity for DWI, which indicated that DWI could be a valuable imaging method for assessing pCR to NACT in breast cancer. An approximately 0.91 AUC value, which was close to 1 (the perfect test result), also indicated an ideal diagnostic performance. The DOR of a test can serve as a single summary measure since it obtains from combining different sensitivity and specificity. It is defined as the ratio of the odds of positivity in disease relative subjects to the odds of positivity in the non-diseased [40]. The DOR value display in a wide range from 0 to infinity. A higher DOR value represents a better ability for the discrimination of the test performance. The outcome of our study has shown that the DOR estimated for DWI was 27.00 (95% CI, 15.60–46.73). This benign high-DOR value indicated that DWI could monitor pCR in NACT accurately.

Although the pooled statistic of our study implied that DWI might accurately detect pCR for breast cancer to NACT, significant heterogeneity in sensitivity (*I*^2^ = 62.7%) and specificity (*I*^2^ = 84.9%) were also noticed. The Spearman correlation coefficients of DWI (0.565, *P* > 0.05) already eliminated the threshold effect had on DWI evaluation. However, the considerable heterogeneity might be attributed to many other factors, such as variations in definition to separate responders from non-responders, variations in the duration of the study population, or differences in pathologic complete response rate or study designs. To reduce the influence induced by these diversities, we carried out several subgroups analyses concerning pCR rate, time duration of study population, and study design. Meta-regression analyses revealed no significant difference among the three subgroups. This finding implied that, although heterogeneity might exist between different studies, results across studies were still comparable with little or no differences outcomes.

Abundant studies have been conducted to evaluate the efficiency of DCE-MRI in diagnosing pathologic response to NACT for patients with breast cancer. Yet, only a few has investigated the DWI diagnostic accuracy for predicting pCR to NACT. Gu et al. [11] suggested that the sensitivity and specificity of DWI were 93 and 85%, respectively. Another meta-analysis, Wu et al. [41] reported a 93% sensitivity and a 82% specificity for DWI evaluation. It seems that both studies have a slightly higher sensitivity and a much higher specificity than our research. However, the two previous analyses only included a small amount of studies which provided DWI data. Gu et al. enrolled eight studies, and Wu et al. had six. Our study analyzed up to 20 groups of DWI data, and this might be the reason causing discrepancy in our result. Interestingly, we can observe in all three studies that the sensitivity of DWI is higher than the specificity. This finding might further prove the hypothesis that DWI could accurately assess pCR in sensitivity. However, it might lack specificity.

Generally, by predicting the outcome and identifying the pCR to NACT treatment, breast cancer patients can avoid inappropriate chemotherapy at early stage as well as additional toxic therapies, and hold a better chance to achieve pCR [42, 43]. Therefore, some researchers argue that it is crucial to find a specific time for DWI evaluation. However, the previous studies and our analysis all failed to find an exact time to perform DWI. The timing of DWI assessment in our research was varied from study to study. Many studies conducted DWI at several time points. Five studies performed DWI after 1 cycle of therapy, three conducted after 2 cycles, and four studies assessed after 3 cycles. The available data were limited and hampered us to perform subgroup analysis. Nevertheless, a pattern can still be observed from the included studies. It seems that the first 3 cycles might be the preferable timing for DWI assessment. This hypothesis needs further approval.

Some limitations should be taken into account in our analysis. First, a majority of the included studies contained a relatively small patients’ population which might weaken the statistical power of the study and might bring about inconclusive and imprecise results. Although quality assessment and publication bias test confirmed that the included studies were eligible, the effect brought by different sample size still could not be neglected. Second, patients with different breast cancer subtypes would be assigned with different treatment regimens which might eventually lead to different pathological responses [24, 25, 33]. Bufi et al. remarked that DWI might achieve a better diagnostic performance in luminal and hybrid tumor subtypes [24]. Another study by Bufi et al. reported that pretreatment ADC was capable of detecting pCR in Triple negative and HER2^+^ tumors [25]. Richard et al. found that luminal A and B subtypes had a lower pretreatment ADC than triple-negative tumors which indicated a superior performance of DWI in the prediction of pCR to NACT in triple-negative tumors [33]. Thus, subgroup analysis based on tumor phenotypes is desirable. However, the limited information, which only three studies had provided data of breast cancer subtypes, prevented us to conduct subgroup analysis on this aspect. Moreover, the definition of pCR could be a reason affecting the diagnostic accuracy test. Since too many various pCR definitions were applied in the included studies, such as Miller-Payne grading system, Mandard’s TRG criteria, Japanese Breast Cancer Society criteria, Chevalier-Sataloff classifications and classification by user-defined, subgroup comparison could not be performed in our study. Yet, although subgroups and threshold effect evaluation has diminished the influence of heterogeneity, the effect of heterogeneity still cannot be eliminated completely. Several variables, regarding treatment regiments of NACT, timing of pCR evaluation, standards and pattern of DWI measurement, and the optimal cut-off values of diagnosis, should be taken into consideration. However, the information retrieved from the included studies were limited and inconsistent regarding the above factors, making it impossible to conduct subgroup analyses to eliminate their effect.

## Conclusion

Despite some limitations, the findings of our study indicate that DWI modality holds a relatively high sensitivity and specificity for the evaluation and prediction of pCR of breast cancer to NACT. The result of our analysis suggests that the application of DWI in combination with other imaging modality may yield greater precision and accuracy in assessing the pCR after NACT.

## Abbreviations

ADC: Apparent diffusion coefficient
AUC: Area under the curve
DCE-MRI: Contrast-enhanced magnetic resonance imaging
DOR: Diagnostic odds ratio
DWI: Diffusion-weighted imaging
FN: False negative
FP: False positive
MRI: Magnetic resonance imaging
NACT: Neoadjuvant chemotherapy
pCR: Pathological complete response
PET/CT: Positron emission tomography-computed tomography
pNR: Pathological non-responders
SROC: Summary receiver operating characteristic curve
TN: True negative
TP: True positive
